# Correction: Anti-TNF and thiopurine therapy in pregnant IBD patients does not significantly alter a panel of B-cell and T-cell subsets in 1-year-old infants

**DOI:** 10.1038/s41424-018-0040-5

**Published:** 2018-07-31

**Authors:** Michael G. Kattah, Jeffrey M. Milush, Trevor Burt, Robert P. McCabe, Michael I. Whang, Averil Ma, Uma Mahadevan

**Affiliations:** 1 Department of Medicine, Division of Gastroenterology, University of California San Francisco, San Francisco, CA USA; 2 Department of Medicine, Division of Experimental Medicine, University of California San Francisco, San Francisco, CA USA; 3 Department of Pediatrics, Division of Neonatology, University of California San Francisco, San Francisco, CA USA; 4 Department of Medicine, Division of Gastroenterology, University of Minnesota, Minneapolis, MN USA

**Correction to:** *Clinical and Translational Gastroenterology* 10.1038/s41424-018-0018-3; published online 3 April 2018

The original version of this article contained an error in Fig. 2, in which part of the text in the legend was omitted. Figure 2 legend should read “Fig. 2. Infants exposed to combination therapy with an anti-TNF agent and an immunomodulator exhibited a trend toward reduced CD27^+^ B cells, switched memory B cells, plasmablasts, IFNγ-producing CD4^+^ and CD8^+^ T cells, and CD4^+^ CCR5^+^ T cells, which did not reach statistical significance. **a** Ranked *p*-values for cell subsets in the three-group analysis comparing CZP vs IFX/ADA vs IFX/ADA + IM. **b**–**g** Median, interquartile ranges, and ranges of frequencies of each cell subset, expressed as a percent of the parent population, are shown. The *p*-value and *q*-value displayed in **b**–**g** were calculated in the three-group analysis. KW Kruskal–Wallis.”

Furthermore, the figure legends were missing for the Supplementary figure files. The HTML has now been updated to include a corrected version of the Supplementary Information.

